# Supplementary material for: Modeling glioblastoma heterogeneity as a dynamic network of cell states
Source: Mol Syst Biol. 2021 Sep 16;17(9):e10105. doi: 10.15252/msb.202010105 (PMC8444284; doi:10.15252/msb.202010105)
Supplement: Supplementary file 5 — Source Data for Figure 3 [file MSB-17-e10105-s001.zip › Figure3A_sourcedata/GSEA_3065/hallmarks_state5.GseaPreranked.1623416302988/HALLMARK_ANDROGEN_RESPONSE.html]

Details for gene set HALLMARK\_ANDROGEN\_RESPONSE[GSEA]

|  || Dataset | state5 |
| Phenotype | NoPhenotypeAvailable |
| Upregulated in class | na\_pos |
| GeneSet | HALLMARK\_ANDROGEN\_RESPONSE |
| Enrichment Score (ES) | 0.28797123 |
| Normalized Enrichment Score (NES) | 1.0962476 |
| Nominal p-value | 0.26640928 |
| FDR q-value | 0.28769475 |
| FWER p-Value | 0.949 |
Table: GSEA Results Summary

  

Fig 1: Enrichment plot: HALLMARK\_ANDROGEN\_RESPONSE      
 Profile of the Running ES Score & Positions of GeneSet Members on the Rank Ordered List

  

| PROBE | GENE SYMBOL | GENE\_TITLE | RANK IN GENE LIST | RANK METRIC SCORE | RUNNING ES | CORE ENRICHMENT || 1 | B2M |  |  | 61 | 0.601 | 0.0362 | Yes |
| 2 | HMGCR |  |  | 79 | 0.562 | 0.0745 | Yes |
| 3 | ACSL3 |  |  | 88 | 0.543 | 0.1125 | Yes |
| 4 | LMAN1 |  |  | 95 | 0.533 | 0.1500 | Yes |
| 5 | ITGAV |  |  | 118 | 0.508 | 0.1839 | Yes |
| 6 | ABHD2 |  |  | 163 | 0.473 | 0.2128 | Yes |
| 7 | SPCS3 |  |  | 183 | 0.454 | 0.2432 | Yes |
| 8 | SCD |  |  | 284 | 0.391 | 0.2599 | Yes |
| 9 | TMEM50A |  |  | 341 | 0.364 | 0.2797 | Yes |
| 10 | SLC38A2 |  |  | 472 | 0.319 | 0.2880 | Yes |
| 11 | DHCR24 |  |  | 795 | 0.242 | 0.2692 | No |
| 12 | SLC26A2 |  |  | 1058 | 0.204 | 0.2544 | No |
| 13 | FADS1 |  |  | 1148 | 0.191 | 0.2581 | No |
| 14 | ELOVL5 |  |  | 1320 | 0.169 | 0.2510 | No |
| 15 | LIFR |  |  | 1520 | 0.150 | 0.2394 | No |
| 16 | HMGCS1 |  |  | 1899 | 0.118 | 0.2055 | No |
| 17 | DNAJB9 |  |  | 1987 | 0.112 | 0.2038 | No |
| 18 | PLPP1 |  |  | 2048 | 0.108 | 0.2048 | No |
| 19 | B4GALT1 |  |  | 2347 | 0.089 | 0.1778 | No |
| 20 | AKAP12 |  |  | 2478 | 0.081 | 0.1690 | No |
| 21 | GNAI3 |  |  | 2504 | 0.079 | 0.1718 | No |
| 22 | SEC24D |  |  | 2586 | 0.074 | 0.1680 | No |
| 23 | AZGP1 |  |  | 2661 | 0.070 | 0.1648 | No |
| 24 | ABCC4 |  |  | 2736 | 0.066 | 0.1612 | No |
| 25 | INSIG1 |  |  | 3055 | 0.049 | 0.1291 | No |
| 26 | ANKH |  |  | 3098 | 0.047 | 0.1277 | No |
| 27 | FKBP5 |  |  | 3209 | 0.041 | 0.1183 | No |
| 28 | PIAS1 |  |  | 3269 | 0.038 | 0.1144 | No |
| 29 | CDC14B |  |  | 3363 | 0.033 | 0.1063 | No |
| 30 | NGLY1 |  |  | 3462 | 0.029 | 0.0974 | No |
| 31 | ELK4 |  |  | 3506 | 0.027 | 0.0945 | No |
| 32 | RPS6KA3 |  |  | 4037 | 0.006 | 0.0354 | No |
| 33 | SRP19 |  |  | 4199 | -0.001 | 0.0175 | No |
| 34 | ZMIZ1 |  |  | 4395 | -0.010 | -0.0037 | No |
| 35 | HOMER2 |  |  | 4586 | -0.018 | -0.0237 | No |
| 36 | APPBP2 |  |  | 4699 | -0.022 | -0.0347 | No |
| 37 | ZBTB10 |  |  | 4886 | -0.029 | -0.0535 | No |
| 38 | PGM3 |  |  | 5211 | -0.041 | -0.0869 | No |
| 39 | STK39 |  |  | 5333 | -0.046 | -0.0972 | No |
| 40 | CAMKK2 |  |  | 5432 | -0.049 | -0.1047 | No |
| 41 | NDRG1 |  |  | 5645 | -0.060 | -0.1242 | No |
| 42 | GSR |  |  | 5778 | -0.065 | -0.1344 | No |
| 43 | PDLIM5 |  |  | 6127 | -0.081 | -0.1676 | No |
| 44 | XRCC5 |  |  | 6246 | -0.087 | -0.1746 | No |
| 45 | ARID5B |  |  | 6331 | -0.092 | -0.1774 | No |
| 46 | IQGAP2 |  |  | 6450 | -0.099 | -0.1836 | No |
| 47 | RAB4A |  |  | 6632 | -0.110 | -0.1961 | No |
| 48 | CCND3 |  |  | 6881 | -0.128 | -0.2147 | No |
| 49 | NCOA4 |  |  | 7044 | -0.141 | -0.2228 | No |
| 50 | MYL12A |  |  | 7289 | -0.166 | -0.2383 | No |
| 51 | TPD52 |  |  | 7309 | -0.168 | -0.2284 | No |
| 52 | UAP1 |  |  | 7314 | -0.168 | -0.2168 | No |
| 53 | IDI1 |  |  | 7337 | -0.172 | -0.2069 | No |
| 54 | UBE2J1 |  |  | 7351 | -0.175 | -0.1959 | No |
| 55 | VAPA |  |  | 7519 | -0.193 | -0.2008 | No |
| 56 | ACTN1 |  |  | 7520 | -0.193 | -0.1870 | No |
| 57 | CENPN |  |  | 7528 | -0.194 | -0.1738 | No |
| 58 | UBE2I |  |  | 7770 | -0.228 | -0.1845 | No |
| 59 | AKT1 |  |  | 7805 | -0.235 | -0.1715 | No |
| 60 | PMEPA1 |  |  | 7830 | -0.241 | -0.1570 | No |
| 61 | CDK6 |  |  | 8156 | -0.310 | -0.1713 | No |
| 62 | SAT1 |  |  | 8162 | -0.314 | -0.1494 | No |
| 63 | PA2G4 |  |  | 8320 | -0.360 | -0.1413 | No |
| 64 | XRCC6 |  |  | 8325 | -0.361 | -0.1159 | No |
| 65 | TSC22D1 |  |  | 8334 | -0.363 | -0.0908 | No |
| 66 | CCND1 |  |  | 8346 | -0.368 | -0.0657 | No |
| 67 | ADRM1 |  |  | 8548 | -0.458 | -0.0554 | No |
| 68 | SMS |  |  | 8834 | -0.690 | -0.0380 | No |
| 69 | DBI |  |  | 8895 | -0.764 | 0.0100 | No |
Table: GSEA details [plain text format]

  

Fig 2: HALLMARK\_ANDROGEN\_RESPONSE: Random ES distribution      
 Gene set null distribution of ES for **HALLMARK\_ANDROGEN\_RESPONSE**
